# Supplementary material for: One-shot learning and behavioral eligibility traces in sequential decision making
Source: eLife. 2019 Nov 11;8:e47463. doi: 10.7554/eLife.47463 (PMC6897511; doi:10.7554/eLife.47463)
Supplement: Transparent reporting form [file elife-47463-transrepform.docx]

***eLife’s* transparent reporting form**

We encourage authors to provide detailed information *within their submission* to facilitate the interpretation and replication of experiments. Authors can upload supporting documentation to indicate the use of appropriate reporting guidelines for health-related research (see [EQUATOR Network](http://www.equator-network.org/%20)), life science research (see the [BioSharing Information Resource](https://biosharing.org/)), or the [ARRIVE guidelines](http://www.plosbiology.org/article/info:doi/10.1371/journal.pbio.1000412) for reporting work involving animal research. Where applicable, authors should refer to any relevant reporting standards documents in this form.

If you have any questions, please consult our Journal Policies and/or contact us: [editorial@elifesciences.org](mailto:editorial@elifesciences.org).

**Sample-size estimation**

- You should state whether an appropriate sample size was computed when the study was being designed
- You should state the statistical method of sample size computation and any required assumptions
- If no explicit power analysis was used, you should describe how you decided what sample (replicate) size (number) to use

Please outline where this information can be found within the submission (e.g., sections or figure legends), or explain why this information doesn’t apply to your submission:

As an estimate of the effect size, we assumed that effect sizes are similar to previous experiments with a very similar experimental set up (Tartaglia, Clarke & Herzog, 2017) and therefore used similar sample sizes. Additionally, we performed a simulated experiment (see Figure 9) using N=10 and N=20 simulated agents with previously published parameter values.

**Replicates**

- You should report how often each experiment was performed
- You should include a definition of biological versus technical replication
- The data obtained should be provided and sufficient information should be provided to indicate the number of independent biological and/or technical replicates
- If you encountered any outliers, you should describe how these were handled
- Criteria for exclusion/inclusion of data should be clearly stated
- High-throughput sequence data should be uploaded before submission, with a private link for reviewers provided (these are available from both GEO and ArrayExpress)

Please outline where this information can be found within the submission (e.g., sections or figure legends), or explain why this information doesn’t apply to your submission:

The experiment was repeated in 3 different conditions: spatial, sound, clip-art, see Figures 2 and 3. Each of these conditions was performed by a different set of participants. The same participant solved the same condition mulitiple times (up to 7 times). On average,participants completed 48.1 episodes in spatial condition , 19.4 episodes in the sound condition and 25.1 episodes in the clip-art condition

Nr. of participants (biological replication) and number of pupil traces (technical replicates) are indicated in Figs. 2e, 2f (behavior), in Fig. 3. (pupil) and in Methods 4.1 Experimental Conditions

The pupil data preprocessing pipeline and outlier rejection is described in the Methods, Section 4.2. Additionally, Figure 7 “Results including low-quality pupil traces” shows the pupil data analysis when all data is included.

**Statistical reporting**

- Statistical analysis methods should be described and justified
- Raw data should be presented in figures whenever informative to do so (typically when N per group is less than 10)
- For each experiment, you should identify the statistical tests used, exact values of N, definitions of center, methods of multiple test correction, and dispersion and precision measures (e.g., mean, median, SD, SEM, confidence intervals; and, for the major substantive results, a measure of effect size (e.g., Pearson's r, Cohen's d)
- Report exact p-values wherever possible alongside the summary statistics and 95% confidence intervals. These should be reported for all key questions and not only when the p-value is less than 0.05.

Please outline where this information can be found within the submission (e.g., sections or figure legends), or explain why this information doesn’t apply to your submission:

Nr. of participants (biological replication) and number of pupil traces (technical replicates) are indicated in Figs. 2e, 2f (behavior), in Fig. 3. (pupil) and in Methods.

Pupil traces were compared using a paired-samples t-test, FDR corrected. P-values are indicated in Figs. 2 and 3 and in the result section of the main text.

The free model-parameters were estimated using the Metropolis-Hastings Markov Chain Monte Carlo (MCMC) algorithm, simulating and collecting a total of 100’000 samples. The Bayesian posterior distribution of the estimation of the free model-parameters is indicated in Fig. 8 and in the main text.

Significance of model selection is established using cross-validation and a Wilcoxon rank-sum test (Result section). Test results and p-values are indicated in Table1 alongside with the Akaike Weights wAIC.

(For large datasets, or papers with a very large number of statistical tests, you may upload a single table file with tests, Ns, etc., with reference to sections in the manuscript.)

**Group allocation**

- Indicate how samples were allocated into experimental groups (in the case of clinical studies, please specify allocation to treatment method); if randomization was used, please also state if restricted randomization was applied
- Indicate if masking was used during group allocation, data collection and/or data analysis

Please outline where this information can be found within the submission (e.g., sections or figure legends), or explain why this information doesn’t apply to your submission:

Participants recruitement and groups are indicated in the Methods section “Experimental conditions”.

**Additional data files (“source data”)**

- We encourage you to upload relevant additional data files, such as numerical data that are represented as a graph in a figure, or as a summary table
- Where provided, these should be in the most useful format, and they can be uploaded as “Source data” files linked to a main figure or table
- Include model definition files including the full list of parameters used
- Include code used for data analysis (e.g., R, MatLab)
- Avoid stating that data files are “available upon request”

Please indicate the figures or tables for which source data files have been provided:

The datasets generated during the current study are available on Dryad, at the following address:

Dryad, Dataset, https://doi.org/10.5061/dryad.j7h6f69
